# Fourteen years of progress testing in radiology residency training: experiences from The Netherlands

**DOI:** 10.1007/s00330-017-5138-8

**Published:** 2017-12-01

**Authors:** D. R. Rutgers, F. van Raamt, W. van Lankeren, C. J. Ravesloot, A. van der Gijp, Th. J. ten Cate, J. P. J. van Schaik

**Affiliations:** 1 Department of Radiology, University Medical Center, Heidelberglaan 100, 3584 CX Utrecht, The Netherlands; 2 Department of Radiology, Gelre Hospital, Apeldoorn, The Netherlands; 3 Department of Radiology, Erasmus University, Rotterdam, The Netherlands; 4 Center for Research and Development of Education, University Medical Center, Utrecht, The Netherlands

**Keywords:** Clinical competence, Educational measurement, Internship and residency, Learning, Radiology

## Abstract

**Objectives:**

To describe the development of the Dutch Radiology Progress Test (DRPT) for knowledge testing in radiology residency training in The Netherlands from its start in 2003 up to 2016.

**Methods:**

We reviewed all DRPTs conducted since 2003. We assessed key changes and events in the test throughout the years, as well as resident participation and dispensation for the DRPT, test reliability and discriminative power of test items.

**Results:**

The DRPT has been conducted semi-annually since 2003, except for 2015 when one digital DRPT failed. Key changes in these years were improvements in test analysis and feedback, test digitalization (2013) and inclusion of test items on nuclear medicine (2016). From 2003 to 2016, resident dispensation rates increased (Pearson’s correlation coefficient 0.74, P-value <0.01) to maximally 16 %. Cronbach´s alpha for test reliability varied between 0.83 and 0.93. The percentage of DRPT test items with negative item-rest-correlations, indicating relatively poor discriminative power, varied between 4 % and 11 %.

**Conclusions:**

Progress testing has proven feasible and sustainable in Dutch radiology residency training, keeping up with innovations in the radiological profession. Test reliability and discriminative power of test items have remained fair over the years, while resident dispensation rates have increased.

***Key Points*:**

*• Progress testing allows for monitoring knowledge development from novice to senior trainee.*

*• In postgraduate medical training, progress testing is used infrequently.*

*• Progress testing is feasible and sustainable in radiology residency training.*

## Introduction

Development of clinical competence in medical trainees can be classified according to the well known four-level pyramid of Miller [1]. The first two levels of competence, which are summarized with the terms ´knows´ and ´knows how´, primarily refer to cognition. They are often assessed with knowledge tests. In residencies, such tests have traditionally been taken in the form of final examinations or modular tests. In The Netherlands, modular testing became part of radiology residency training in the 1980s. Residents had to complete ten modular tests on various subspecialty domains. Tests were distributed over the total training period of 5 years, and started in the beginning of residency. A major limitation was that novices were forced to study subspecialty domains in depth, although they had little radiological experience in this field. Also, once a subspecialty test was passed, it generally was not revisited later during residency. Such one-point measurements can hamper knowledge retention and may not be representative of knowledge development of trainees [2]. Another limitation was that large and small subspecialty domains were valued equally since each had a comparable knowledge test in the curriculum. A type of knowledge assessment that may not suffer from these limitations is progress testing [3, 4].

Progress testing as a model of assessment was developed in the 1970s independently in the medical schools of the University of Maastricht in The Netherlands and the University of Missouri in the USA [5, 6]. In progress testing, all learners are simultaneously given the same test, irrespective of their training stage. The test is repeatedly given during the curriculum, usually two to four times per year, which allows for monitoring the expected improvement in knowledge from novice to senior trainee. The test is based on the learning objectives of a given curriculum. All relevant disciplines within that curriculum are represented in the test, according to a test blue-print that defines the contribution of each discipline [3]. Individual test items may vary from test to test, but the blue-print remains stable as long as learning objectives do not change. Progress tests have been implemented by many medical schools, but are not common in postgraduate medical training [3]. In 2003, a progress test was implemented in radiology residency training in The Netherlands, replacing previous modular testing. This test, the Dutch Radiology Progress Test (DRPT), has been evolving ever since.

The purpose of the present study was to describe the development of progress testing in Dutch radiology residency training from its start in 2003 up to 2016. We assessed key changes and events in the DRPT during this period, determined resident participation and dispensation rates for the test, and calculated test reliability and discriminative power of test items over the years.

## Materials and methods

### Set-up of the DRPT

When introduced in 2003, the DRPT was designed as a semi-annual knowledge test on all subspecialty domains of radiology. It was organized under the responsibility of the Examination Committee of the Radiological Society of The Netherlands. Because test results were merely used for feedback purposes, without any pass/fail decision, it was a formative assessment. It was a required test during all training years, signifying a total of ten DRPTs during the 5 years of radiology residency. Residents could apply at the Examination Committee for dispensation from participation for various reasons, such as attendance of a course or congress, holidays, leave, health issues and other circumstances in personal life. In 2003 and 2004, residents were exempted from participation if they had passed the required modular tests in the years before introduction of the DRPT. Although the exact number was difficult to assess in retrospect from our files, we estimate that over 30 residents were exempted from participation in April 2003, declining to a small number of residents in October 2004.

The DRPT started as a paper-and-pencil test with 200 true/false/don´t-know test items, without radiological images. The ´don´t-know´ answer option was included to discourage guessing and to simulate authentic clinical practice in which residents can consult a supervisor [7, 8]. Formula scoring (i.e. one point for a correct answer, no point for a ‘don’t-know’ choice, and a penalty of minus one point for an incorrect answer) was used to calculate test scores. This type of scoring is thought to reduce random error by minimizing guessing [9].

### Data collection and statistical analysis

The ethical review board of The Netherlands Association for Medical Education approved conduct of this study (dossier number 883).

We reviewed all DRPTs that were conducted since 2003, both the tests themselves and all relevant correspondence within the Examination Committee referring to the tests. We assessed which key changes and events occurred in the DRPT throughout the years, and we described its practice in 2016. Key changes were defined as changes and events that affected the form, structure or carrying out of the test. For each we described when, what and why it changed or occurred.

We assessed the number of participating residents in each test. Also, we assessed the number of residents who were given dispensation from participation, relative to the total number of residents who were eligible for participating. Pearson´s correlation was calculated between dispensation rate and time period after the introduction of the DRPT. Test reliability was estimated with Cronbach’s alpha. To assess the degree of discriminative power of test items, we calculated their R_ir_-value (item-rest correlation). This value indicates the correlation between a given item score and the score on the remaining test items of the same test. It is considered an objective measure for the degree to which a test item can discriminate strongly performing from weakly performing candidates. A high R_ir_-value indicates that candidates who performed well on the test as a whole performed well on that particular test item, whereas candidates who performed poorly on the test as a whole scored poorly on that test item. The lower the R_ir_-value, the weaker this relation becomes. If the R_ir_-value is zero, there is no discrimination. A negative R_ir_-value indicates that candidates who performed well on the test as a whole performed poorly on that test item, and vice versa. Thus, items with negative R_ir_-values discriminate between candidates in an opposite, unintended way. Such items challenge the quality of the test. Therefore, we assessed the number of test items with a R_ir_-value < 0 in each DRPT, relative to the total number of items in a test. In addition, the mean R_ir_-value was calculated for test items in each DRPT, and Pearson´s correlation was calculated between mean R_ir_-value and time period after the introduction of the DRPT. Normality of parameters was investigated with the Kolmogorov-Smirnov test. A P-value < 0.05 was considered statistically significant.

## Results

### Development of the DRPT since 2003

The first paper-and-pencil DRPT was completed by 175 residents in Utrecht, a centrally located Dutch city, in April 2003. Table 1 gives an overview of key changes and events that have occurred since then. From October 2004 on, the DRPT included image-based test items. From 2007 on, statistical analysis of the DRPT was performed by the Center for Research and Development of Education of the University Medical Center in Utrecht, independently of the Examination Committee of the Radiological Society of The Netherlands. Because the practice of radiology rapidly evolved from an analogue to a digitally oriented profession, the need for a digital DRPT increased. After a 2-year preparation period, the first digital DRPT was taken in 383 residents on two test locations (Amsterdam and Utrecht) in April 2013. Digital testing allowed for more advanced image-based test items, such as volumetric image-based items and drag and drop items. In October 2013, the don´t-know answer option was abandoned as research on the DRPT showed that this option weakened the test validity [10]. In addition, it was decided to use number-right scoring instead of formula-scoring. In number-right scoring, a score is given for correct answers without penalties for wrong answers. It was considered to better fit the main DRPT purpose of assessing knowledge levels in residents [10], and to be potentially less biased in favor of candidates with more risk-taking test behavior [9]. In April 2014, we reduced the total number of DRPT test items from 200 to 180 since advanced digital image-based items in the test can be time-consuming for participants when they have to scroll through volumetric datasets or place markers in drag and drop items. The digital DRPT of autumn 2015 failed due to computer problems on the day of examination. This led to the re-introduction of a back-up paper-based test. In 2016, test items on nuclear medicine were included in the DRPT, as a result of the merge of the residency programmes for radiology and nuclear medicine into one combined training programme in The Netherlands in 2015.

**Table 1:** Key changes and events in the Dutch Radiology Progress Test from 2003 to 2016

Year	Time of year	Change, event	Cause, background
2003	Spring	Introduction of the paper-and-pencil DRPT with 200 true/false/don´t-know test items, without radiological images	Limitations of previous modular knowledge tests in radiology training in The Netherlands
2004	Autumn	Introduction of image-based test items, comprising up to one-fifth of the total number of test items	Better representation of clinical radiological practice by means of image-based test items
2007	Autumn	Statistical analysis of the DRPT conducted by the Center for Research and Development of Education of the University Medical Center in Utrecht	Improvement of test analysis and test feedback
2009	Spring	Removal of radiological physics as a subspecialty domain from the DRPT, and introduction of a modular test on radiological physics in the resident training programme	Low scores on test items on radiological physics in the DRPT
2010	Autumn	One-time experimental set-up of the DRPT with two groups of participants	Investigation of the value of the don´t-know answer option in the DRPT
2013	Spring	Replacement of the paper-and-pencil test by a digital DRPT in two test locations with 200 test items of which one-sixth was image-based (2D); back-up paper-based test available	Better representation of clinical radiological practice by means of digital images; opportunity of more advanced image-based test items than in the paper-and-pencil test
	Autumn	Digital DRPT conducted at one test location, centrally located in the country, instead of two locations	Less complex logistics in organizing the DRPT
		Introduction of other test items than true-false items, such as multiple choice, long-list menu and drag and drop items	Reducing the effect of guessing and better representation of clinical radiological practice
		Introduction of volumetric image-based test items	Better representation of clinical radiological practice than with 2D image-test items only
		Abandoning of the don´t-know answer option	Statistical analysis showed that the don´t-know answer option weakened the validity of the DRPT [10]
		Introduction of number-right scoring instead of formula-scoring	Number-right scoring was considered to better fit the main test purpose of estimating knowledge level of residents [10], and to potentially be less biased [9]
2014	Spring	Reduction of the total number of test items from 200 to 180	Advanced digital image-based test items are time-consuming for participants, and necessitated a reduction in the number of items that can be posed in a fixed examination time frame
		Increase of proportion image-based test items to approximately one quarter of the total number of test items	Better representation of clinical radiological practice
	Autumn	Abandoning of the back-up paper-based test	Successful implementation of the previous two digital DRPTs
		Proportion image-based test items maximized at one third of the total number of test items	Avoiding too many time-consuming image-based test items, within fixed examination time frame
2015	Autumn	Failure of digital DRPT	Technical computer-related problems at test location
2016	Spring	Re-introduction of back-up paper-based test	Back-up test available in case of technical failure of the digital DRPT
		Test items on nuclear medicine included in the DRPT	Merging of the residency training programmes of radiology and nuclear medicine in The Netherlands, as a result of which the learning objectives of the curriculum changed

*DRPT* Dutch Radiology Progress Test

### Participation rate, test reliability and discriminative power

**Fig. 1:**
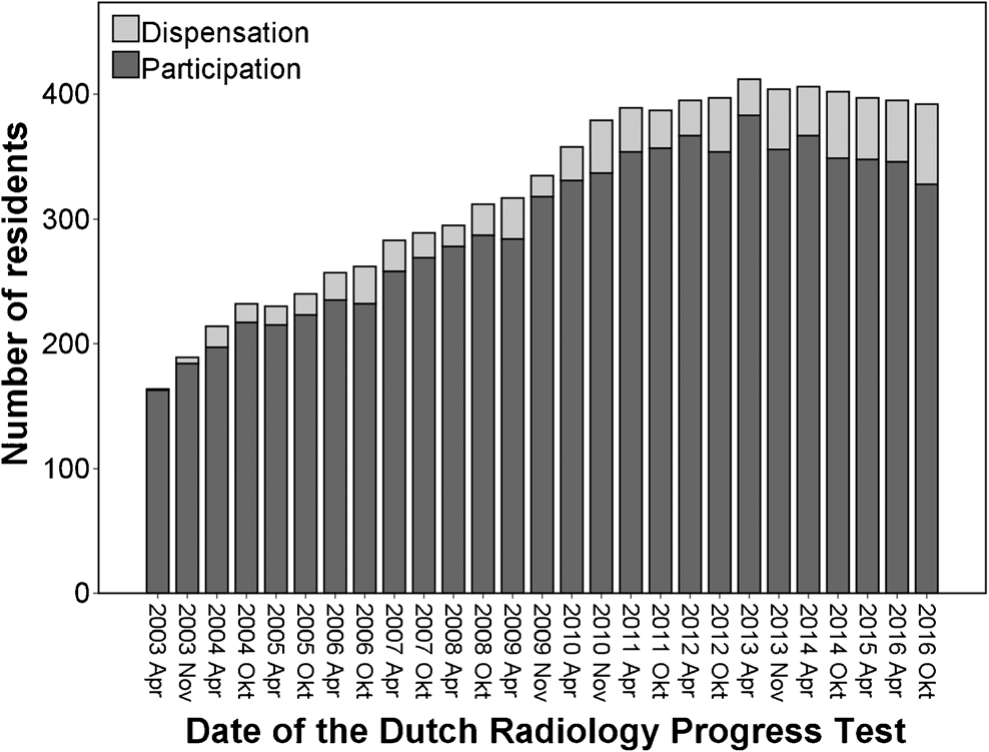
Number of residents participating in or given dispensation from participating in the Dutch Radiology Progress Tests from 2003 to 2016

**Fig. 2:**
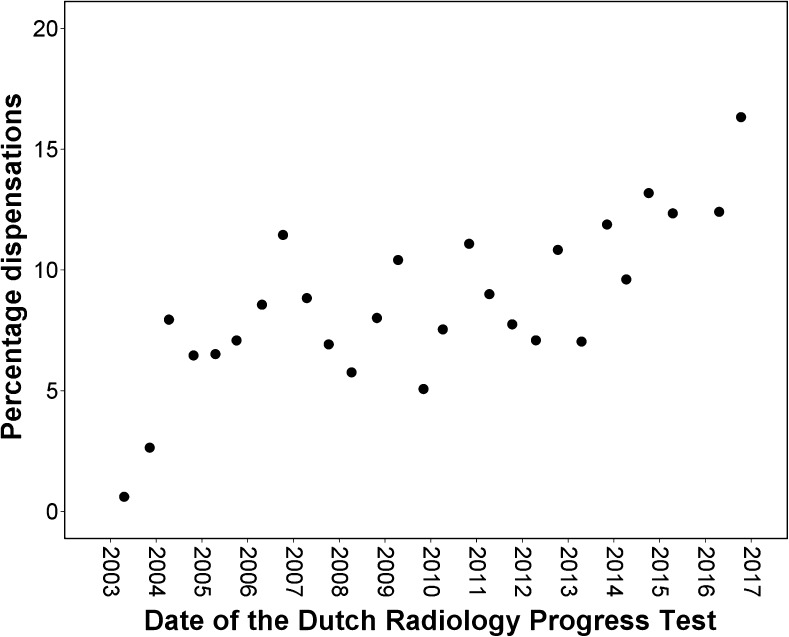
Percentage of residents who were given dispensation from participating in the Dutch Radiology Progress Test, relative to the total number of residents who were eligible for participating, from 2003 to 2016

The investigated parameters were normally distributed. The number of residents participating in the DRPT increased from 175 in 2003 to a maximum of 383 in April 2013, declining to 328 in October 2016 (Fig. 1). The number of residents that were given dispensation from participating varied from one in 2003 to 64 in 2016. The total number of residents, either participating or given dispensation, increased from 2003 (164) to April 2013 (412). The percentage of residents who were given dispensation, relative to the total number of residents eligible for participating, is given in Fig. 2. This percentage varied from 1 % to 16 %, and showed a statistically significant increase since the introduction of the DRPT (Pearson’s correlation coefficient 0.74, P < 0.01).

Test reliability and discriminative power of test items are given in Table 2. Cronbach´s alpha and R_ir_-values could not be reconstructed for the period 2003–2004 and 2003–2005, respectively, because source data were no longer available. In 2005–2016, Cronbach´s alpha varied between 0.83 and 0.93. In 2006–2016, the mean R_ir_-value of DRPT test items varied between 0.15 and 0.25 and was not significantly correlated with the time period that had elapsed since the introduction of the DRPT (Pearson’s correlation coefficient -0.10, P=0.66). The percentage test-items with R_ir_-values < 0 varied between 4 % and 11 %, relative to the total number of test items. In 19 out of 21 DRPTs (90 %) of which R_ir_-values were available, ≤ 10 % of test items had a negative R_ir_-value.

**Table 2:** Reliability, mean R_ir_-value of test items and number of test items with negative R_ir_-value in the semi-annual Dutch Radiology Progress Test from 2005 to 2016

Year	Month	Number of participating residents	Maximum number of test items	Cronbach´s alpha ^a^	Mean R_ir_-value (SD) of test items	Number of test items with negative R_ir_-value (% of max. number of test items)
2005	April	215	200	0.88	n.a.	n.a.
	October	223	200	0.88	n.a.	n.a.
2006	April	235	200	0.92	0.23 (0.11)	7 (4 %)
	October	232	200	0.87	0.17 (0.12)	15 (8 %)
2007	April	258	200	0.89	0.19 (0.13)	13 (7 %)
	October	269	200	0.88	0.18 (0.14)	22 (11 %)
2008	April	278	200	0.89	0.19 (0.13)	16 (8 %)
	October	287	200	0.91	0.21 (0.15)	17 (9 %)
2009	April	284	200	0.92	0.23 (0.14)	12 (7 %)
	November	318	200	0.90	0.20 (0.13)	15 (8 %)
2010	April	331	200	0.91	0.21 (0,15)	16 (8 %)
	November	337	200	0.84, 0.83 ^b^	0.19 (0.13), 0.15 (0.13) ^b^	9 (5 %)
2011	April	354	200	0.91	0.22 (0.14)	12 (6 %)
	October	357	200	0.91	0.21 (0.16)	15 (8 %)
2012	April	367	200	0.93	0.25 (0.15)	14 (7 %)
	October	354	200	0.92	0.24 (0.15)	10 (5 %)
2013	April	383	200	0.92	0.24 (0.14)	12 (6 %)
	November	356	200	0.86	0.17 (0.12)	17 (9 %)
2014	April	367	180	0.89	0.20 (0.13)	15 (8 %)
	October	349	180	0.90	0.21 (0.14)	11 (6 %)
2015	April	348	180	0.87	0.18 (0.13)	18 (10 %)
	October ^c^	-	-	-	-	-
2016	April	346	180	0.85	0.17 (0.14)	19 (11 %)
	October	328	180	0.87	0.18 (0.13)	14 (8 %)

^a^After exclusion of inadequate test items

^b^One-time experimental set-up with two groups of participants to investigate the value of the don´t-know answer option in the DRPT

^c^Digital DRPT of October 2015 failed due to technical reasons

*DRPT* Dutch Radiology Progress Test, *R*_*ir*_ item-rest-correlation, *SD* standard deviation, *n.a.* not assessed

### Current practice of the DRPT

The purpose of the DRPT is to assess the level and progression of radiological knowledge in radiology residents in The Netherlands in a uniform way during their training programme. The test is based on the learning objectives of the national radiology residency training programme, as defined by the Radiological Society of The Netherlands, and covers the eight subspecialty domains of this programme (Table 3). DRPT procedures, responsibilities and protocols on adverse test events are documented in examination regulations.

**Table 3:** Subspecialty domains in the Dutch Radiology Progress Test (2016)

Subspecialty domain	Total number of test items	Number of test items without images	Number of image-based test items
Cardiac and thoracic radiology	36	24	12
Abdominal radiology	36	24	12
Interventional radiology	12	8	4
Nuclear medicine and molecular radiology	12	8	4
Neuroradiology and head-and-neck radiology	30	20	10
Musculoskeletal radiology	30	20	10
Breast radiology	12	8	4
Paediatric radiology	12	8	4
Total	180	120	60

The DRPT is taken semi-annually (generally in April and October) at one central test location in Amsterdam. Radiology residents from all 28 training locations in The Netherlands, both academic and non-academic teaching hospitals, are automatically signed up for each DRPT during their 5-year residency. The DRPT is a required test for all residents, but they can apply to the Examination Committee of the Radiological Society of The Netherlands for dispensation from participating in a test, with written support of their local programme director. The most common reasons for dispensation are leave, health issues and attendance of courses and congresses. No separate re-examination is scheduled for residents who are given dispensation for a test. These residents are automatically signed up for the next regular DRPT. In the formative setting of the DRPT, in which test results are merely used for feedback purposes, dispensation for an individual test has no direct consequence for possible future graduation from the training programme. The DRPT is performed digitally with software (www.vquest.nl) that has been developed specifically for image-based testing [11]. The DRPT consists of 180 items to be answered within 2 h and 45 min. A sealed back-up paper-based test is available during examination. It has been drafted separately from the digital test items and consists of 150 test items among which 30 are 2D image-based items. If not opened, the paper test can serve as a back-up test for several years.

The content of the DRPT is drafted by the Examination Committee of the Radiological Society of The Netherlands. The committee is composed of at least nine radiologists from both teaching and non-teaching hospitals throughout The Netherlands, who collectively cover all DRPT subspecialty domains. Multiple rounds of feedback are completed during preparation of each DRPT. Firstly, two radiologists prepare test items for one subspecialty domain, providing each other with feedback. Several radiologists prepare items for more than one domain. Secondly, items are presented in writing to all other radiologists of the Examination Committee for written feedback. Finally, all test items are reviewed in a joint review meeting in which items can be viewed ´live´ with the test software. A new set of test items is drafted for each DRPT. After a test has been taken, the Examination Committee obtains feedback through standardized evaluation forms from residents and through statistical test analysis, after which it decides if test items should be removed. Finally, the Examination Committee assesses the DRPT end scores, including overall scores and scores on subspecialty domains. Individual results are communicated to each participating resident and to his or her programme director. For benchmarking purposes, the latter is also informed on the overall performance of residents in his or her training location, compared with the other training locations in The Netherlands.

## Discussion

In the present study, we describe our experiences with formative progress testing in Dutch radiology residency training since 2003. The main findings are twofold. Firstly, the DRPT has been successfully implemented and developed since 2003, keeping up with innovations of the radiological profession in The Netherlands in terms of digitalization and subspecialty developments. Secondly, this process has been accompanied by a fair quality of the DRPT, as evidenced by sufficient test reliability and a relatively low percentage of indiscriminative test items, while there was a trend to higher dispensation rates over the years.

Knowledge of radiology residents is often assessed through modular tests or final or intermediate exams [12–14]. Progress testing has a number of advantages over these forms of knowledge assessment [3]. A progress test is designed to test the end objectives of a given curriculum, rather than intermediate courses and levels, which may encourage deeper learning styles. By repeatedly testing the same domain of knowledge, long-term knowledge retention is promoted. In addition, progress testing reduces the need for re-examinations because consecutive tests provide the opportunity to repeatedly demonstrate growth of knowledge. In The Netherlands, progress tests have been developed in a number of postgraduate training programmes such as in obstetrics and gynaecology and in general practice [15, 16]. Implementation of progress testing may be hampered by several disadvantages of this form of assessment, such as the high level of central organization required and the need for resources for test development and scoring [3]. Wrigley et al. have suggested a systematic framework for development of a progress test [17]. It describes four main phases, including test construction, test administration, results analysis and review, and feedback to stakeholders. In our experience, the elements of test administration and those of test analysis and review are reasonably well covered in the DRPT. Test construction and feedback to stakeholders may be improved on several elements such as item bank construction and feedback to teachers and overview committees. These may be a subject of future development of the DRPT.

With respect to test reliability, a Cronbach´s alfa of 0.70–0.79 may be considered acceptable in formative assessment such as the DRPT [18]. Higher values have been achieved in the DRPT throughout the years, despite the changes in the test. As a rule-of-thumb, we aim to construct DRPTs in which the proportion of items with negative R_ir_-values is not greater than 10 % of the total number of test items. We found that in 19 out of 21 DRPTs we were able to meet this criterion. Besides reliability, validity is a fundamental element in the evaluation of measurement instruments [19, 20]. We did not specifically investigate validity in the present study, but a previous study provided support for the validity of the DRPT [8]. In that study, progress test scores, defined as the correct-minus-incorrect score over all test items in a DRPT, were compared between different years of residency. Progress test scores on knowledge items increased significantly from training year 1 to year 3 (tested with one-way analysis-of-variance). After the fourth year of residency, no significant increase in scores was found. Dispensation rates were not analysed in that study. The reported increase in knowledge scores in the first 3 years of residency supports the construct validity of the test. Validity is no fixed quality of the DRPT, but should be re-assessed in the future. In order to maintain adequate reliability and validity in future DRPTs, a wide range of factors is important [4]. For example, good reliability depends on sufficient test length, item difficulty and item discrimination. These latter factors should be continuously monitored by item analysis. To maintain good test validity, it has to be repeatedly analysed whether the test still fits the learning objectives of the training programme and whether participants improve on the test while progressing through their training programme. One of the main changes in the DRPT was the introduction of digital testing in 2013. Digital image-based test-items can be time consuming when participants have to scroll through volumetric datasets or place markers in drag and drop test items. To anticipate this effect, we have chosen to reduce the number of test items in the DRPT rather than to increase the examination time. This did not lead to a reduction of test reliability below the aforementioned threshold of 0.70–0.79 for Cronbach´s alfa in formative assessment [18].

The total number of residents who were eligible for participating in the DRPT increased between 2003 and 2013 from 164 to 412. This reflects the government-driven increase in radiology residents in The Netherlands in these years. Although the DRPT is required for all residents, dispensation is given in individual cases, generally differing from test to test. We observed a trend towards higher dispensation rates for residents over the years. This may be related to changes in the population and activities of residents. For example, the attendance of courses and congresses may have increased over the years, or the amount of leave may have grown with changing demographics among the resident group. Likewise, we cannot exclude that a certain habituation towards the DRPT occurred over the years, which may have changed the attitude towards participation.

This study has a number of limitations. Firstly, it is a retrospective study in which changes and events in the development of the DRPT are investigated after the fact. Secondly, we did not systematically conduct interviews with residents and programme directors from the introduction of the DRPT on. However, through national meetings on the quality of the radiology training programme and through test evaluation forms, we regularly received feedback on the DRPT. Thirdly, the described practice of radiological progress testing applies specifically to The Netherlands. Its relatively small geographical scale facilitates simultaneous testing of all residents from the country in one test location under equal conditions. Still, postgraduate radiological progress testing can be organized in other countries as well. In our experience, a number of conditions are important for successful implementation: collaboration among programme directors who share a common objective of implementing progress testing, a central organization team that is supported by resources from the national radiological society and radiological training institutions throughout the country, and support by experts on medical education.

In conclusion, progress testing has proven a feasible and sustainable way of formative knowledge testing in radiology residency training in The Netherlands. It has moved from a paper-and-pencil test to a digital test with volumetric image-based test items, keeping up with innovations of the radiological profession.

## Abbreviations

DRPT: Dutch Radiology Progress Test
